# Papillary Thyroid Carcinoma with Terminal Immune Exhaustion Phenotype Correlates with Increased Risk of Lymph Node Metastasis: An Exploratory Study Combining Flow Cytometry and TCGA

**DOI:** 10.3390/cancers18172873

**Published:** 2026-09-05

**Authors:** Shixu Wang, Huizhu Cai, Ruochan Zhang, Kun Chen, Wan Liu, Zehao Huang, Dangui Yan, Chunfeng Qu, Zhengjiang Li

**Affiliations:** 1 Department of Head and Neck Surgery, National Cancer Center/National Clinical Research Center for Cancer/Cancer Hospital, Chinese Academy of Medical Sciences and Peking Union Medical College, Beijing 100021, China; wangshixu@cicams.ac.cn (S.W.); tyr1573@163.com (W.L.); yandangui@cicams.ac.cn (D.Y.); 2 Department of Gastroenterological Surgery, Peking University People’s Hospital, Beijing 100044, China; caihuizhu@pkuph.edu.cn; 3 Department of Immunology, National Cancer Center/National Clinical Research Center for Cancer/Cancer Hospital, Chinese Academy of Medical Sciences and Peking Union Medical College, Beijing 100021, China; seira328@163.com (R.Z.); chenkun@cicams.ac.cn (K.C.); 4 Department of Thyroid and Breast Surgery, Peking University First Hospital, Beijing 100035, China; hzh518518@yeah.net

**Keywords:** papillary thyroid carcinoma, lymph node metastasis, tumor microenvironment, terminal immune exhaustion

## Abstract

Background: Papillary thyroid carcinoma (PTC) is the most common thyroid malignancy, with lymph node metastasis (LNM) being a key predictor of recurrence and poor prognosis. Preoperative detection of LNM remains challenging due to the limitations of imaging modalities, leading to inadequate surgical resection in 20–30% of patients. While immune checkpoint molecules have been implicated in PTC progression, the heterogeneity of CD8^+^ T cell exhaustion subsets and their specific association with LNM remain poorly defined. In this study, we aimed to perform an exploratory characterization of the distinct immune landscape of PTC prone to LNM, with a focus on terminal immune exhaustion, in order to generate hypotheses for improved risk stratification and therapeutic strategies. Methods: Fresh PTC tissues from 40 patients (22 LNM-positive and 18 LNM-negative) were analyzed via flow cytometry (FCM) to quantify immune cell subsets, inflammatory cytokines, and chemokines. Immunohistochemistry (IHC) validated CD45^+^ immune cell infiltration. Transcriptomic and clinical data from 448 PTC patients in The Cancer Genome Atlas (TCGA-PTC) cohort were used for bioinformatic analysis consistent with the observed phenotype, including Gene Set Variation Analysis (GSVA) of terminal exhaustion gene signatures. Results: LNM-positive PTC exhibited a unique inflammatory milieu with significantly elevated IL-6, IL-1ra, CCL5, and IL-9 levels (all *p* < 0.05) in tumor interstitial fluid. FCM analysis revealed that LNM-positive PTC had increased infiltration of total CD45^+^ immune cells, CD3^+^ T cells, and CD3^+^CD8^+^ T cells (all *p* < 0.05). Critically, terminally exhausted PD-1^hi^TIM-3^+^ CD8^+^ T cells were significantly enriched in LNM-positive PTC (*p* = 0.022) and positively correlated with extrathyroidal extension (*p* = 0.044). Additionally, LNM risk was associated with increased CD4^+^ regulatory T (Treg) cell frequency (*p* = 0.023) and elevated CTLA-4 expression on CD4^+^ T cells (*p* = 0.047). In TCGA-PTC validation, the terminal exhaustion gene signature was predominantly enriched in LNM-positive (*p* < 0.0001) and advanced-stage PTC (*p* < 0.001) and strongly correlated with *BRAF* mutation (predominantly V600E) (*p* < 0.0001)—the most common oncogenic driver in aggressive PTC. Conclusions: Our findings suggest a terminal immune exhaustion phenotype (characterized by PD-1^hi^TIM-3^+^ CD8^+^ T cells and Treg enrichment) as a potential key feature associated with LNM-prone PTC. This phenotype shows consistency across clinical samples and TCGA datasets, linking *BRAF* mutation (predominantly V600E) to immune suppression and metastatic potential. These insights provide a novel exploratory immune-based biomarker for LNM risk stratification and support the potential of combining anti-PD-1/TIM-3 therapy with *BRAF* inhibitors for high-risk PTC, which should be confirmed in future studies.

## Simple Summary

Papillary thyroid carcinoma (PTC) frequently metastasizes to lymph nodes, yet reliable preoperative biomarkers are lacking. This study investigated the immune landscape of PTC prone to lymph node metastasis (LNM) using flow cytometry, immunohistochemistry, and cytokine profiling, complemented by TCGA transcriptomic analysis. We found that LNM-positive PTC exhibited a terminal immune exhaustion phenotype characterized by enrichment of PD-1^hi^TIM-3^+^ CD8^+^ T cells and regulatory T cells, alongside elevated pro-inflammatory cytokines. TCGA data further supported the association between terminal exhaustion signatures and advanced disease. Our findings offer new immune-related markers for risk evaluation and provide insights for developing combined immunotherapy for high-risk patients, which may help other researchers to design further clinical and experimental studies.

## 1. Introduction

Global thyroid cancer incidence has increased by ~5% annually in recent decades, with PTC accounting for 80–90% of cases [1,2]. While PTC generally has a favorable prognosis, LNM occurs in 30–50% of patients and is associated with a 2- to 3-fold higher recurrence rate and reduced disease-free survival [3,4]. Ultrasound and computed tomography (CT) have limited sensitivity for detecting occult LNM, leading to understaging and incomplete lymph node dissection [5,6]. Thus, identifying reliable biomarkers for LNM risk is crucial to optimize surgical planning and reduce recurrence.

The tumor microenvironment (TME) plays a pivotal role in PTC progression, with immune cell infiltration being a key determinant of metastatic potential [7]. CD8^+^ cytotoxic T cells are critical for antitumor immunity, but persistent antigen stimulation drives their differentiation into exhausted subsets characterized by impaired cytotoxicity and overexpression of checkpoint molecules [8]. Recent studies have highlighted the heterogeneity of exhausted CD8^+^ T cells; for example, stem-like PD-1^int^TCF1^+^ cells retain proliferative potential and responsiveness to immunotherapy, while terminally exhausted PD-1^hi^TIM-3^+^ cells are functionally irreparable and associated with poor outcomes in solid tumors [9]. However, in PTC, most studies have focused on total PD-1+ CD8^+^ T cells without distinguishing exhaustion subsets [10,11], and their specific role in LNM remains unclear.

Regulatory T (Treg) cells, classically defined by CD4^+^CD25^hi^CD127^lo^FOXP3^+^ expression, suppress effector T cell function and promote immune evasion [12]. In this study, Treg cells were phenotypically identified as CD4^+^CD25^hi^CD127^lo^ cells without FOXP3 confirmation (see Methods). Previous reports show increased Treg infiltration in PTC, but the correlations with LNM and crosstalk with exhausted CD8^+^ T cells are not fully elucidated [13,14]. Additionally, *BRAF V600E* mutation—present in 40–60% of PTC—drives tumor proliferation and is associated with aggressive features [15]. Emerging evidence suggests that *BRAF V600E* may modulate the TME by upregulating immune checkpoint molecules [11,16], but its link to terminal immune exhaustion and LNM is unproven. Previous studies have established important links between the immune microenvironment and PTC biology. Some studies have demonstrated that immune infiltration patterns in PTC correlate with tumor invasion, histopathologic aggressiveness, and *BRAF* mutation status [17,18]. Additionally, other studies have linked *BRAF*(*V600E*)/PD-L1 co-expression and PD-L1 expression to clinicopathological aggressiveness, recurrence risk, and metastatic behavior in differentiated thyroid cancer [19,20].

In this study, we integrated FCM, IHC, cytokine profiling, and TCGA transcriptomic analysis to characterize the immune landscape of LNM-prone PTC. We hypothesized that terminal immune exhaustion (PD-1^hi^TIM-3^+^ CD8^+^ T cells) and Treg enrichment constitute a distinct phenotype associated with LNM. Our findings provide novel insights into the immunopathogenesis of PTC metastasis and identify potential biomarkers for risk stratification and targeted immunotherapy.

## 2. Materials and Methods

### 2.1. Patients and Study Design

This retrospective study was conducted at the National Cancer Center/Cancer Hospital, Chinese Academy of Medical Sciences. Consecutive treatment-naive PTC patients who underwent partial/total thyroidectomy and lymph node dissection between June and August of 2023 were enrolled. Inclusion criteria were as follows: (1) histopathologically confirmed PTC; (2) no prior head and neck surgery/radiotherapy; (3) no distant metastasis or other malignancies; and (4) complete clinical and follow-up data. Exclusion criteria were as follows: (1) preoperative immunotherapy/chemotherapy and (2) inadequate tissue samples for FCM/IHC. All patients underwent central lymph node dissection, and therapeutic lateral lymph node dissection was performed for patients with cytologically confirmed lateral LNM (NCCN Guidelines for Thyroid Carcinoma, 2023). LNM was defined via histopathological examination, and TNM staging followed the AJCC 8th edition. This study was approved by the ethics committee of the Cancer Hospital of Chinese Academy of Medical Sciences (reference number: NCC2023C-281). Informed consent was obtained from all participants.

### 2.2. Flow Cytometry (FCM)

Tumor tissues were collected after the tumors were surgically removed and soaked in DMEM containing 2% fetal bovine serum. Within 30 min after tissue collection, the single-cell suspension of each independent tumor tissue was prepared by using a tissue digestion solution containing collagenase IV, as we reported previously [21]. The cells were stained with fluorochrome-conjugated antibodies (CD45, CD3, CD8, CD25, CD127, PD-1, TIM-3, and CTLA-4) with a standard laboratory protocol, as we reported previously [22]. Data were acquired on an LSR-II (BD, San Jose, CA, USA) and analyzed with the FlowJo software (version 10.8.1, TreeStar Inc., Ashland, OR, USA). PD-1^hi^ and PD-1^int^ subsets were distinguished based on fluorescence intensity relative to fluorescence-minus-one (FMO) controls and isotype staining. PD-1^hi^ was defined as cells with fluorescence intensity exceeding the upper quartile of the total PD-1^+^ population within each sample [23]. Regulatory T cells were phenotypically defined as CD3^+^CD4^+^CD25^hi^CD127^lo^ cells; intracellular FOXP3 was not assessed in the flow cytometry panel. The antibodies used are provided in Supplementary Table S1.

### 2.3. Immunohistochemistry (IHC)

Standard laboratory protocols were used for immunohistochemistry staining, as we reported previously [21]. The paraffin-embedded tissues were cut into 4 μm sections. The slides were incubated with the primary antibody to human CD45 (clone 4E9B2, Proteintech) at 4 °C overnight and visualized with a DAB stain system (Zhongshan Jinqiao, Beijing, China). Upon H&E staining, tumor-infiltrating immune cells were identified as basophilic cellular aggregates within the tumor stroma, distinct from thyroid follicular epithelial cells. Slides were scanned using the Aperio ScanScope software (version 10.2, Aperio Technologies, Vista, CA, USA). The primary antibody was omitted for the negative controls. Three representative randomly chosen fields at 200× magnification per slide were used for the evaluation of positive cells. The cell counts were recorded as cells/mm^2^, and the average value was adopted.

### 2.4. Cytokine and Chemokine Quantification

Cytokine profiling was performed on a subset of 13 samples (6 N0, 7 N1) for which sufficient tumor interstitial fluid was available. Tumor interstitial fluid was prepared, and total protein was measured as we reported previously [22]. Cytokines and chemokines (IL-6, IL-1ra, CCL5, IL-9, IL-10, and IL-8) were quantified using the Luminex Multi-factor Detection Platform following the manufacturer’s protocol (kit catalog No.M500KCAF0Y, Bio-Rad Laboratories, Hercules, CA, USA) on the Luminex-200 instrument (Luminex Corporation, Austin, TX, USA). The levels of specified inflammatory cytokines or chemokines were calculated based on the total protein of interstitial fluid.

### 2.5. Bioinformatic Analysis

Transcriptomic (RNA-seq) and clinical data for 448 PTC patients (TCGA-PTC) were downloaded from the Genomic Data Commons (GDC) Portal (https://portal.gdc.cancer.gov/) on 20 June 2023. A terminal exhaustion gene signature was constructed based on the following previously validated markers: PDCD1 (PD-1), HAVCR2 (TIM-3), CD8A, GZMA, PRF1, TIGIT, LAG3, and TOX. Gene Set Variation Analysis (GSVA) scores were calculated using the GSVA R package (v1.46.0) to quantify signature enrichment. GSVA scores represent relative enrichment of the gene signature within each sample and were interpreted as continuous variables. Higher values indicate greater relative enrichment of signature genes at the top of the sample-wise expression ranking. Given the relative nature of GSVA scores, we focused on between-group comparative differences rather than absolute threshold values. *BRAF* mutation data were extracted from TCGA mutation annotations, with V600E as the primary variant of interest.

### 2.6. Statistical Analysis

GraphPad Prism 9.0 was used for statistical analysis. Categorical variables were compared using chi-squared or Fisher’s exact tests. Continuous variables were analyzed with unpaired Student’s *t*-tests (normal distribution) or Mann–Whitney U tests (non-normal distribution). Normality was assessed using the Shapiro–Wilk test. Given the exploratory nature of this study, *p*-values were not adjusted for multiple comparisons; the findings should be interpreted accordingly. Nonparametric tests (Mann–Whitney U) were applied when distributional assumptions were violated. Correlations were assessed using Pearson’s or Spearman’s rank correlation coefficients. *p* < 0.05 was considered statistically significant.

## 3. Results

### 3.1. Clinicopathological Characteristics of the Study Cohort

**Figure 1:**
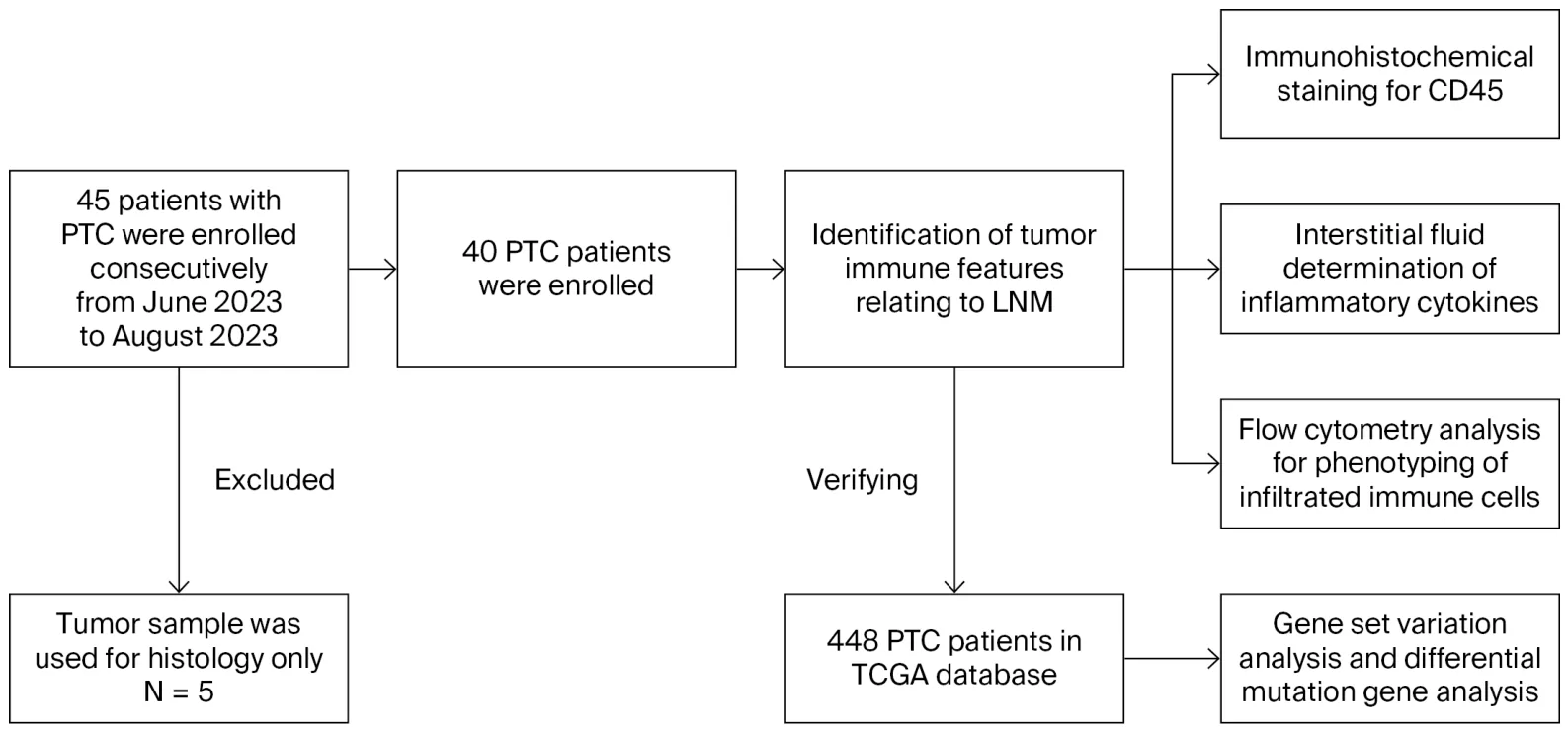
Flow diagram of study design. PTC, papillary thyroid carcinoma; TCGA, The Cancer Genome Atlas.

In 2023, the study consecutively enrolled 45 patients with clinically diagnosed PTC. Tumor tissues from five patients were excluded due to insufficient material for flow cytometry or immunohistochemistry. A total of 40 PTC patients were finally included in the current study (Figure 1). Histological examination after surgery confirmed that 22 patients had PTC with LNM and 18 patients had PTC without LNM. In the current analysis, the cases of PTC with LNM included both N1a, which is defined as metastasis to level VI or VII lymph nodes, and N1b, which is defined as metastasis to lateral neck lymph nodes or retropharyngeal lymph nodes. The patients diagnosed with PTC with LNM (N1) were significantly younger than those with PTC without LNM (N0) (36 ± 7 vs. 46 ± 13 years, *p* = 0.004). Among the 22 LNM-positive patients, 12 had N1a (central compartment) and 10 had N1b (lateral compartment) disease. The median number of dissected lymph nodes was 24, and the median number of metastatic lymph nodes was 8. No significant difference was observed between the N1 and N0 patients in gender, tumor histological types, tumor size, presence of thyroiditis, and benign nodules in peritumoral tissues (Table 1).

### 3.2. PTC Samples with LNM Present a Higher Leukocyte Density

**Figure 2:**
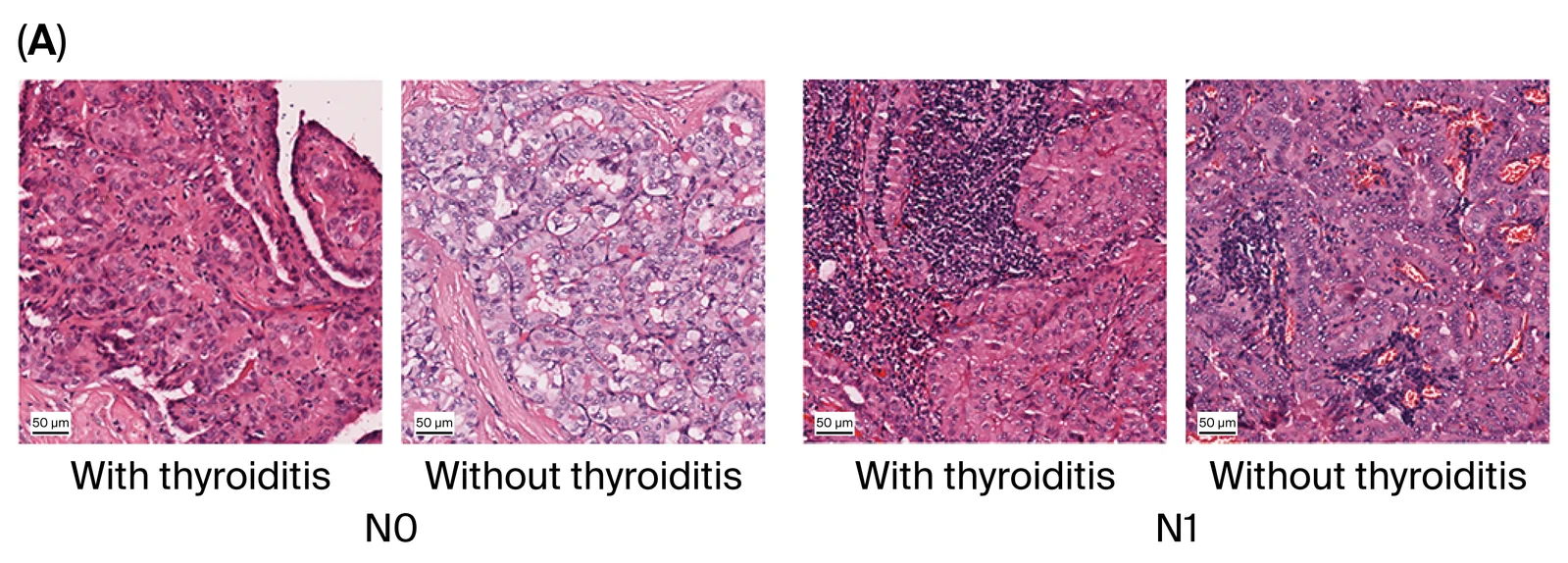

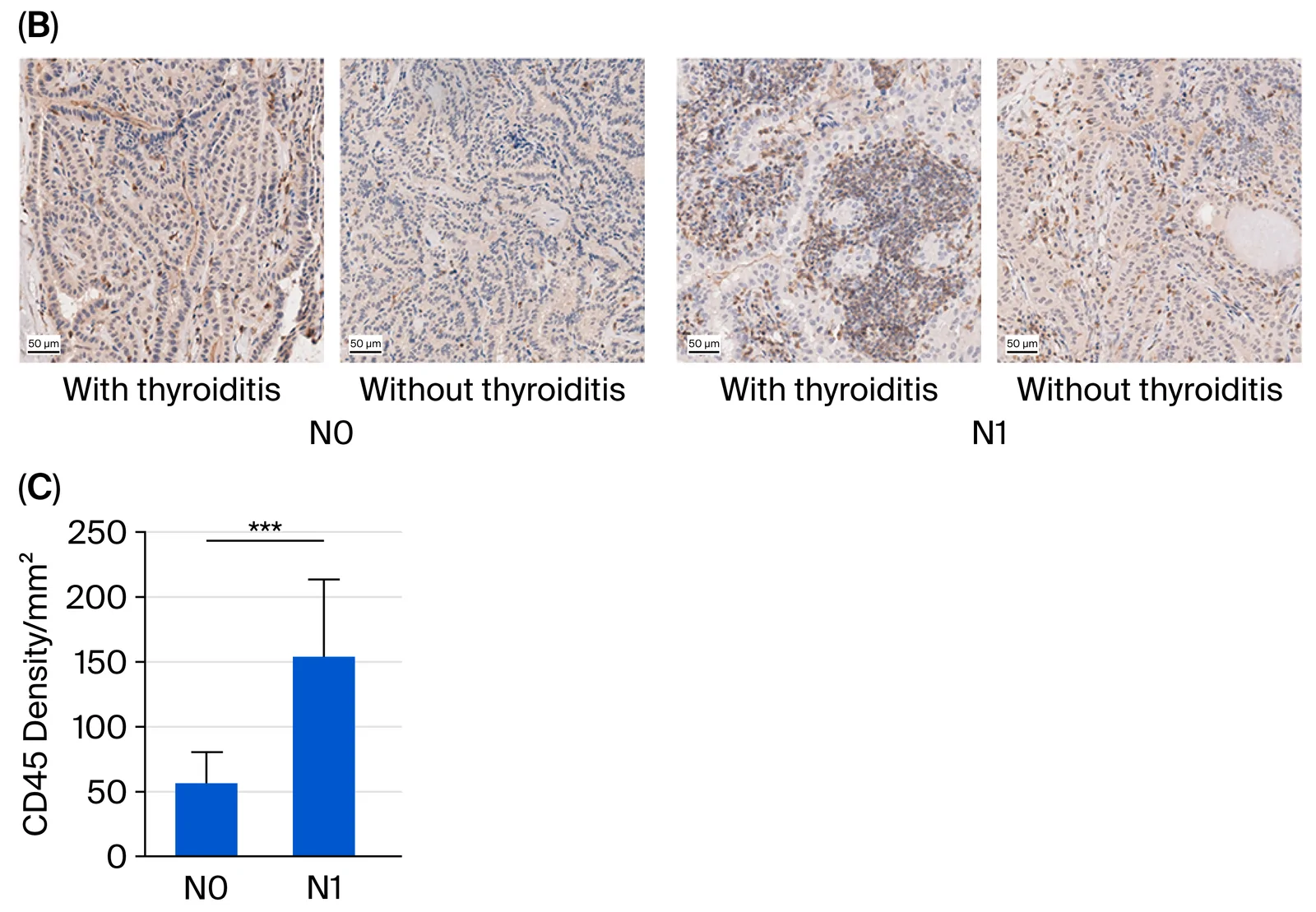
Tumor-infiltrating immune cells in PTC and correlation with LNM. (**A**) Representative images of H&E staining of PTC samples with (N1) or without LNM (N0). (**B**) Representative CD45 immunohistochemistry staining of N1 or N0 tumor tissues. (**C**) Quantification of CD45^+^ cells infiltrating the tumor tissues of N0 (*n* = 18) and N1 (*n* = 22). Data are shown as mean ± SD. *** *p* < 0.001, examined using a two-tailed version of Student’s *t*-test.

Histological examination showed that the PTC tumors of patients with LNM (N1) presented a greater degree of inflammatory infiltration than those of patients without LNM (N0), regardless of the presence or absence of thyroiditis (Figure 2A). Tumor-infiltrating immune cells were identified on H&E as basophilic cellular aggregates in the tumor stroma, distinct from thyroid follicular epithelial cells. For confirmation of the inflammatory infiltration, we performed immunohistochemistry to identify the presence of CD45^+^ total immune cells (Figure 2B). The density of CD45^+^ cells in PTC patients with LNM (N1) was higher than that in PTC patients without LNM (N0) (*p* < 0.001, Figure 2C). Quantification was performed across all samples regardless of thyroiditis status, as both the N0 and N1 groups contained cases with and without concurrent thyroiditis.

### 3.3. PTC Samples with LNM Display Unique Inflammatory Milieu

**Figure 3:**
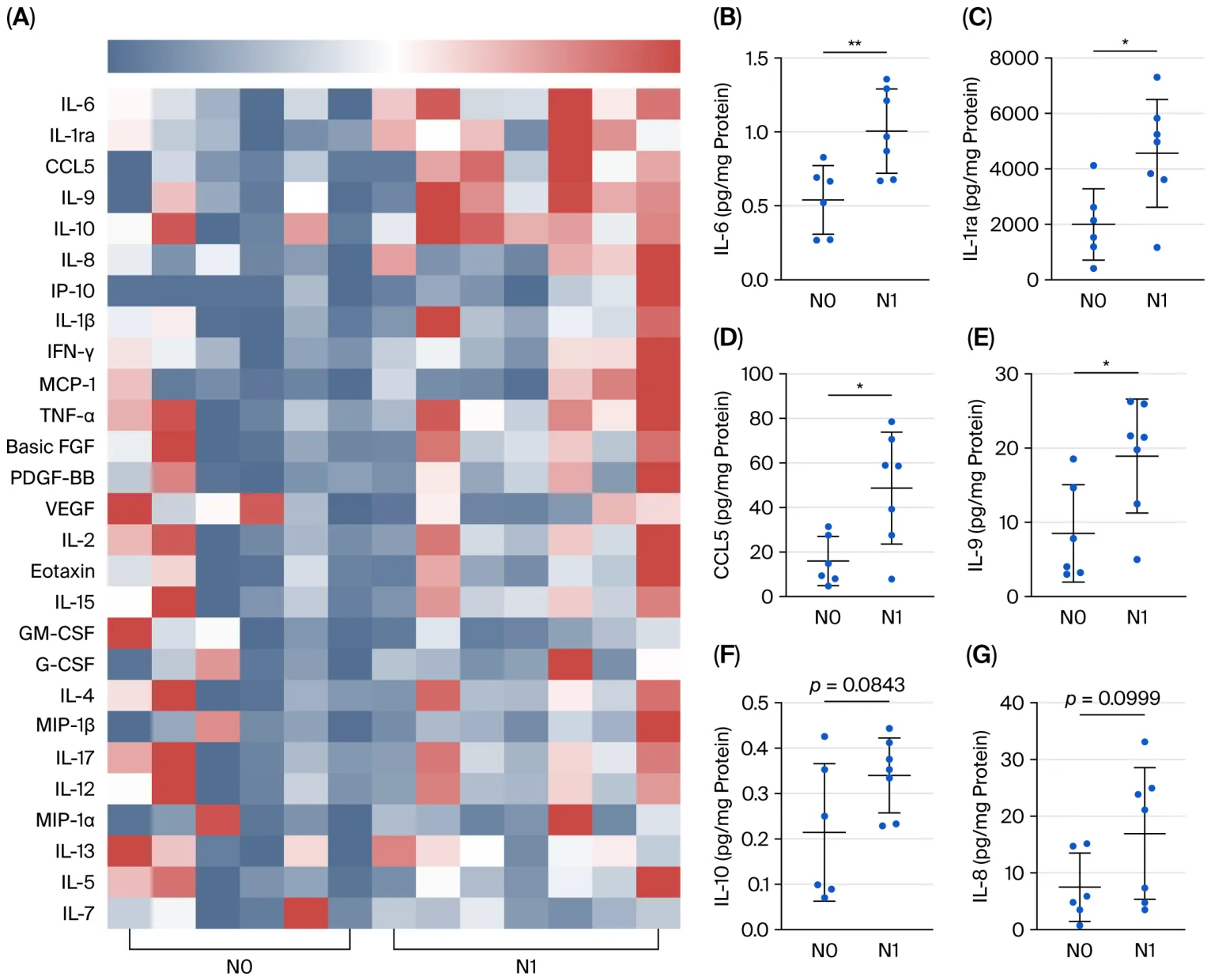
Expression of inflammatory cytokines and chemokines in PTC tumor tissues. (**A**) Heat map illustrating the relative expression of inflammatory cytokines and chemokines presented in tumor intercellular fluid of PTC sample with (N1, *n* = 7) or without LNM (N0, *n* = 6). (**B**–**G**) Concentration of IL-6 (**B**), IL-1ra (**C**), CCL5 (**D**), IL-9 (**E**), IL-10 (**F**), and IL-8 (**G**) in the tumor intercellular fluid. Data are shown as the mean ± SD. * *p* < 0.05 and ** *p* < 0.01, examined using a two-tailed version of Student’s *t*-test.

To potentially reflect the TME character of PTC, we quantified the inflammatory cytokines and chemokines in six PTC samples without LNM (N0) and seven samples with LNM (N1). PTC samples with LNM expressed different inflammatory cytokines and chemokines compared to those without LNM (Figure 3A). The tumor tissue with LNM (N1) presented higher levels of IL-6 (*p* = 0.009), IL-1ra (*p* = 0.013), CCL5 (*p* = 0.020), and IL-9 (*p* = 0.024) than those without LNM (N0) (Figure 3B–E). The levels of IL-10 (*p* = 0.084) and IL-8 (*p* = 0.099) in PTC samples with LNM (N1) were not statistically significantly different from those without LNM (N0) (Figure 3F,G), both of which have been reported to play immunosuppressive roles.

### 3.4. PTC Samples with LNM Present a Greater Infiltration of CD3^+^CD8^+^ T Cells

**Figure 4:**
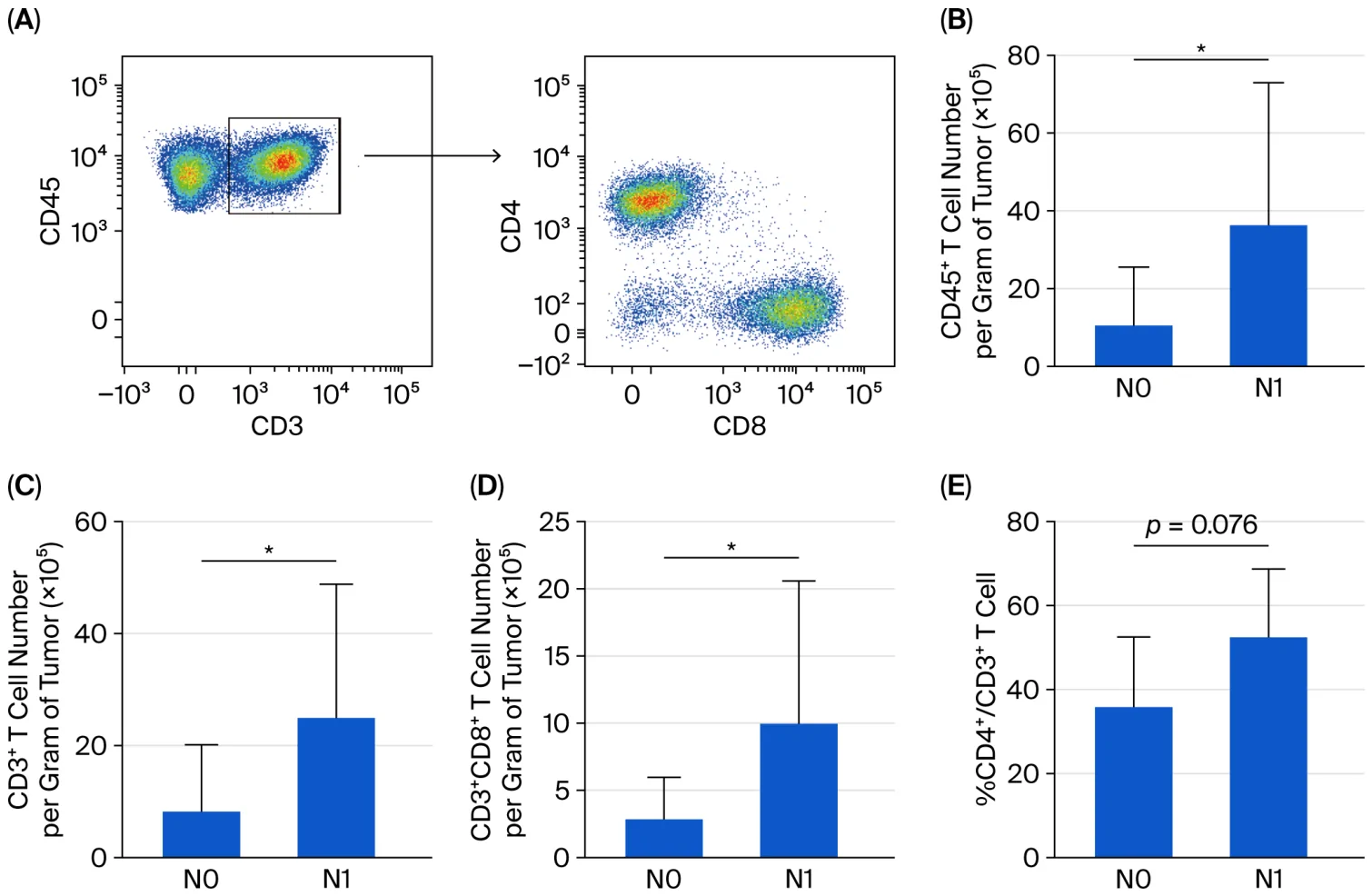
Infiltration of CD3^+^CD8^+^ cytotoxic T cells in PTC and correlation with LNM. (**A**) Representative flow cytometric plots showing the population of CD45^+^ cells, CD3^+^ T cells, and CD3^+^CD8^+^ T cells. (**B**–**D**) Comparison of CD45^+^ cell, CD3^+^ T cell, and CD3^+^CD8^+^ T cell counts in tumor tissue via FCM analyses. (**E**) Bar graph indicating the average percentage of CD4^+^ among CD3^+^ T cells. *n* = 18 in N0; *n* = 22 in N1. Student’s *t*-test was applied. Data are shown as the mean ± SD. All reported *p*-values are two-sided (* *p* < 0.05).

Different types of immune cells within the PTC tissues were further analyzed using FCM (Figure 4A). Tumor tissues with LNM (N1) had greater numbers of CD45^+^ immune cells (*p* = 0.013, Figure 4B) and CD3^+^ T cells (*p* = 0.016, Figure 4C) than PTC samples without LNM (N0). Analysis of different T cell subsets indicated that PTC samples with LNM (N1) had more CD3^+^CD8^+^ T cells than those without LNM (N0) (*p* = 0.016, Figure 4D). Additionally, the infiltrated proportion of CD4^+^ T cells was higher in the PTC samples with LNM (N1) than in those without LNM (N0) (*p* = 0.076, Figure 4E). Given the overlapping error bars and limited sample size, these differences, while statistically significant, should be interpreted with caution regarding biological effect size.

### 3.5. PTC Samples with LNM Had More Exhausted PD-1^+^CD8^+^ T Cells

**Figure 5:**
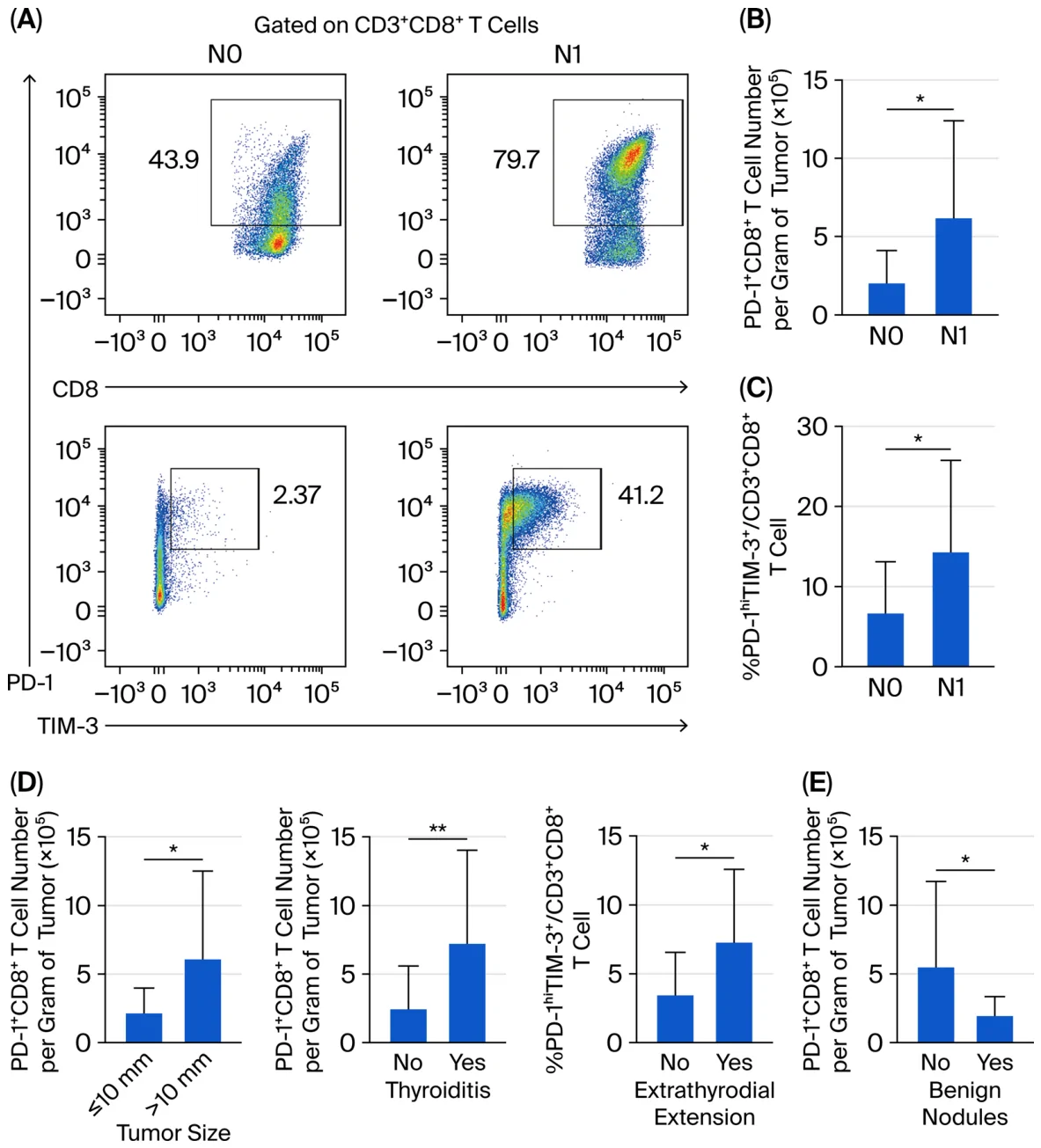
Intra-tumoral PD-1^+^CD8^+^ T cell infiltration indicated tumor progression. (**A**) Representative flow cytometric plots displaying the percentage of intra-tumoral PD-1^+^CD8^+^T cells and PD-1^+^TIM-3^+^CD8^+^ T cells. (**B**) Bar graph indicating the average count of PD-1^+^CD8^+^ T cells per gram of tumor tissue. (**C**) Bar graph indicating the average percentage of PD-1^hi^TIM-3^+^ among CD3^+^CD8^+^ T cells. (**D**,**E**) Bar graph indicating the average count of PD-1^+^CD8^+^ T cells and frequency of PD-1^hi^TIM-3^+^ among CD3^+^CD8^+^ T cells in tumor tissues with different pathological characteristics. *n* = 18 in N0; *n* = 22 in N1. Student’s *t*-test was applied. Data are shown as the mean ± SD. All reported *p*-values are two-sided (* *p* < 0.05; ** *p* < 0.01).

We further analyzed the exhaustion status of CD8^+^ T cells within tumor tissue (Figure 5A). Increased numbers of exhausted PD-1^+^CD8^+^ T cells (*p* = 0.016, Figure 5B), particularly terminally exhausted CD8^+^ T cells, which are defined as PD-1^hi^TIM-3^+^ cells [23], were positively related to PTC with LNM (*p* = 0.022, Figure 5C). Further analysis indicated that the number of infiltrated PD-1^+^CD8^+^ T cells was positively associated with larger tumor size (*p* = 0.043) and concurrent thyroiditis in peritumoral tissues (*p* = 0.007). Increased numbers of PD-1^hi^TIM-3^+^ cells were positively associated with the presence of tumor cell extrathyroidal extension (*p* = 0.044) (Figure 5D). Notably, the presence of benign nodules in the peritumoral tissues was negatively related to the infiltrated number of PD-1^+^CD8^+^ T cells in tumor tissues (*p* = 0.042, Figure 5E). No relationship was found in nerve invasion or blood vessel invasion. Given the overlapping error bars and limited sample size, these associations, while statistically significant, should be interpreted with caution.

### 3.6. Relationship Between Intra-Tumoral CD4^+^ T Cells and LNM

**Figure 6:**
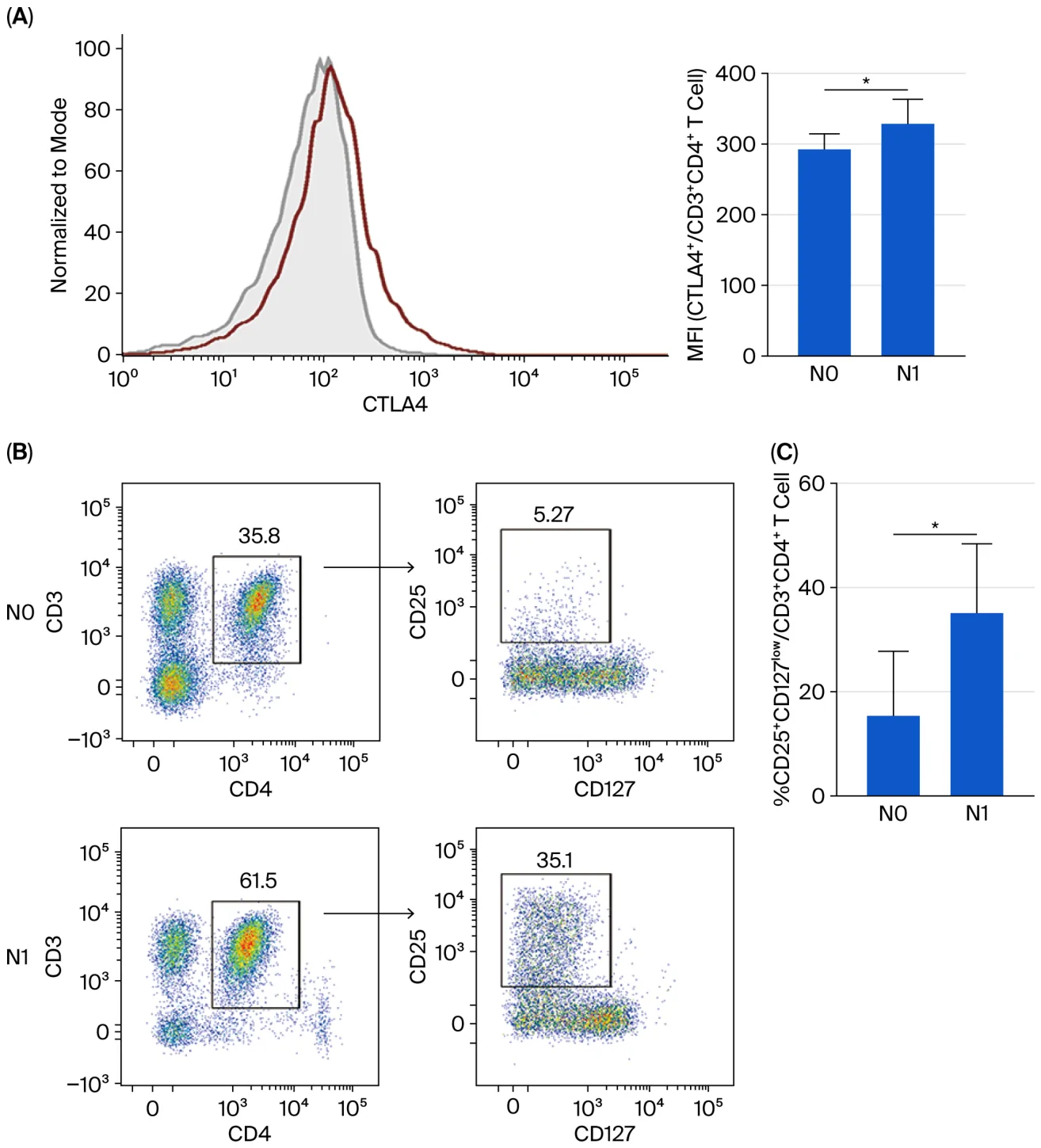
CD3^+^CD4^+^ T cell and Treg infiltration in tumor-indicated LNM. (**A**) Bar graph indicating the expression of CTLA-4 in CD3^+^CD4^+^ T cells analyzed by FCM. Representative histogram shown. (**B**) Representative flow cytometric plots showing CD25^hi^CD127^lo^ T cell infiltration. (**C**) Bar graph indicating the frequency of CD25^hi^CD127^lo^ among CD3^+^CD4^+^ T cells in patients with LNM. *n* = 18 in N0; *n* = 22 in N1. Student’s *t*-test was applied. Data are shown as the mean ± SD. All reported *p*-values are two-sided (* *p* < 0.05).

It has been reported that CTLA-4 is a critical immune-suppressive marker mainly expressed on CD4^+^ Treg cells [24]. As indicated by the mean fluorescence intensity (MFI), the CD4^+^ T cells in the PTC samples with LNM (N1) displayed higher surface expression of CTLA-4 than those without LNM (N0) (*p* = 0.047, Figure 6A). Based on the high expression of CD25 and low expression of CD127 for defining Treg cells [12], we detected an elevated frequency of Treg cells among the total CD3^+^CD4^+^ T cells, and the elevation was significantly associated with LNM (*p* = 0.023, Figure 6B,C). While statistically significant, the modest difference in CTLA-4 MFI (Figure 6A) and the overlapping distributions should be interpreted cautiously given the limited sample size and exploratory nature of this study.

### 3.7. An Exhausted Immune Feature Presented in PTC Patients in the TCGA Cohort with LNM and Tumor Progression

**Figure 7:**
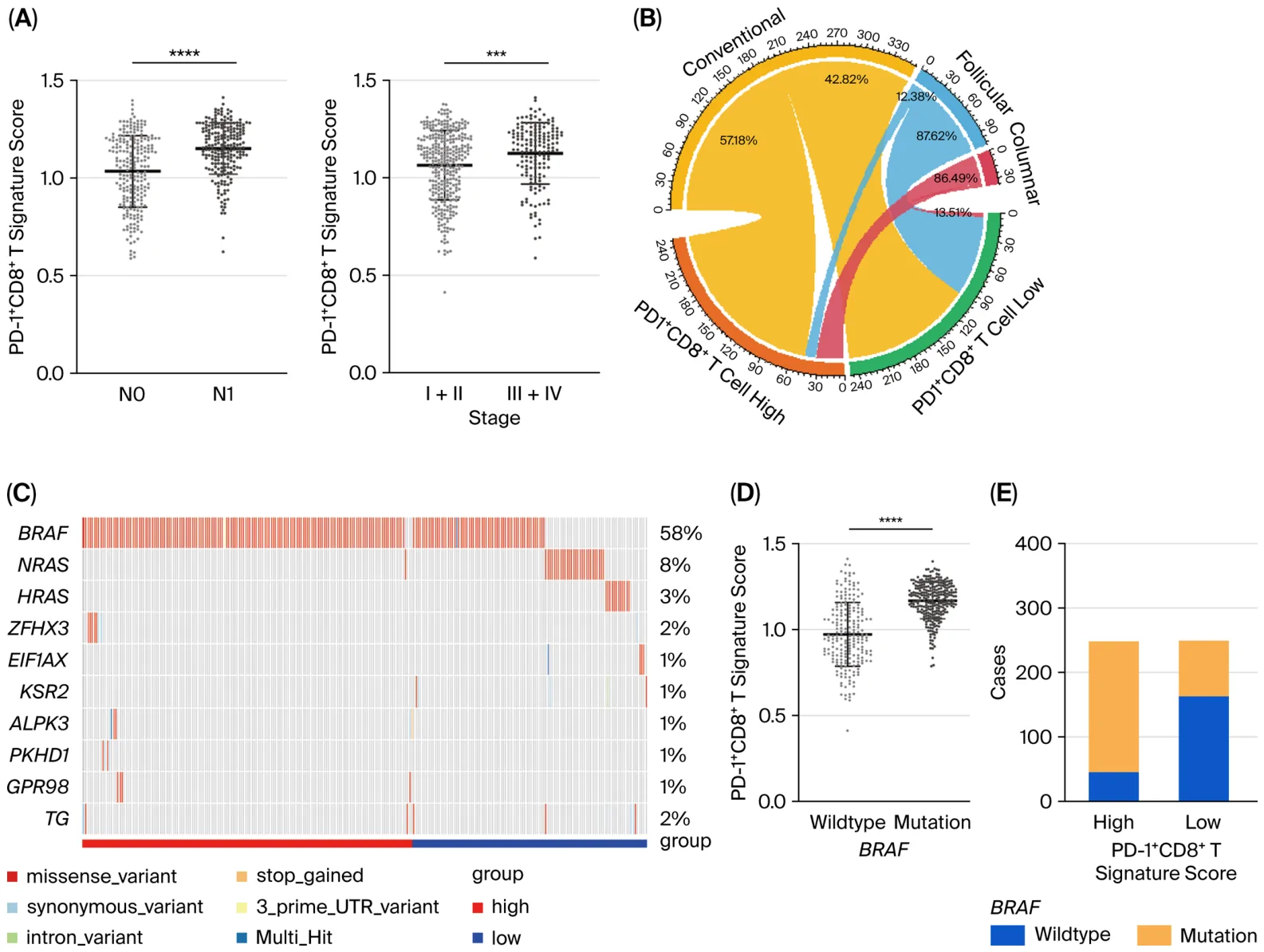
Bioinformatics characteristics of papillary thyroid carcinoma patients with LNM in the TCGA cohort. (**A**) Comparison of terminal exhaustion signature score with LNM and AJCC stage. (**B**) Relationship between terminal exhaustion signature score and pathology subtype of PTC. (**C**) Differential mutation analysis of high- and low-terminal-exhaustion enrichment groups and only specimens with gene mutations are shown. (**D**) Comparison of terminal exhaustion signature score with *BRAF* mutation. (**E**) Comparison of frequency of *BRAF* gene mutation with terminal exhaustion signature score (*** *p* < 0.001; **** *p* < 0.0001).

To validate the immune feature of PTC with LNM, we downloaded the transcriptomic and clinical data of 448 TCGA-PTC samples. Bioinformatic analysis of the PTC samples indicated that the terminal exhaustion signature was significantly enriched in the N1 patients (*p* < 0.0001, Figure 7A). The scores of terminal exhaustion signature in tumor tissues of advanced stage (stages III and IV) were significantly higher than those of early-stage (stages I and II) tumors (*p* < 0.001, Figure 7A). We divided the TCGA-PTC samples into two groups based on median GSVA scores of terminal exhaustion signature, high (*n* = 224) and low (*n* = 224). According to the tumor pathological subtypes, the terminal exhaustion signature was significantly higher in the columnar cell subtype, which is generally believed to have a poor prognosis (Figure 7B) [11].

We also analyzed the relationship between somatic gene mutations and the enrichment of terminal exhaustion signature. In the TCGA-PTC cohort, *BRAF* gene mutation occurred most frequently, detected in 58% of tumors and mainly missense mutations (Figure 7C). The scores of terminal exhaustion signature in the *BRAF*-mutated tumors were significantly higher than in the tumors carrying wild-type *BRAF* (*p* < 0.0001, Figure 7D). Notably, the tumors with high terminal exhaustion scores presented a higher frequency of *BRAF* gene mutation (80.6%) than those with low terminal exhaustion scores (33.3%) (*p* < 0.0001, Figure 7E). Although the absolute difference in mean GSVA scores between N0 and N1 patients was modest, the highly significant *p*-value (*p* < 0.0001) and the large sample size of the TCGA cohort (*n* = 448) support the biological relevance of this differential enrichment.

## 4. Discussion

This study suggests a terminal immune exhaustion phenotype as one of the key features associated with LNM in PTC, supported by integrated analysis of clinical samples and TCGA data. Our major findings are threefold: (1) LNM-positive PTC harbors a unique TME with elevated pro-inflammatory cytokines (IL-6 and CCL5) and enhanced immune cell infiltration; (2) terminally exhausted PD-1^hi^TIM-3^+^ CD8^+^ T cells and Treg enrichment are specific hallmarks associated with LNM-prone PTC; and (3) the terminal exhaustion signature is supported by transcriptomic analysis of TCGA-PTC and strongly correlates with *BRAF* mutation (predominantly V600E).

Insufficient resection can lead to residual tumor in regional lymph nodes, resulting in disease recurrence and requiring secondary surgery. In the current study, we characterized the immune features of PTC that are prone to lymph node metastasis (LNM) by analyzing the infiltration of multiple types of immune cells in the freshly removed tumor tissues. The PTC tissues with LNM presented higher levels of inflammatory cytokine IL-6 and chemokine CCL5 in the interstitial fluid and greater numbers of total immune cells than those without LNM. PTC tumor extrathyroidal extension was positively related to increased infiltration of terminally exhausted PD-1^hi^TIM-3^+^ T cells. LNM risk was positively correlated with the frequency of CD4^+^ Treg cells in tumor tissues. The findings were supported by transcriptomic analysis of the TCGA-PTC cohort, in which the terminally exhausted T cell gene signature was mainly detected in the tumor tissues with LNM and in progressive tumors. The tumor tissues of PTC with LNM carried a terminally exhausted immune feature and were associated with tumor progression.

Tumor-infiltrating cytotoxic CD8^+^ T cells can specifically suppress the progression of tumors but transition to a state of “exhaustion” or “dysfunction” under continuous stimulation by tumor antigens. Exhausted CD8^+^ T cells are characterized by impaired cytotoxicity, decreased production of pro-inflammatory cytokines, and overexpression of multiple inhibitory receptors accompanied by transcriptional and epigenetic changes [25,26]. The presence of CD8^+^ T cell exhaustion, with the expression of T cell inhibitory receptors, has been reported in multiple types of malignant tumors, such as melanoma, non-small cell lung cancer, hepatocellular carcinoma, and ovarian cancer. Co-expression of PD-1 and Tim-3 serves as a marker of the most dysfunctional tumor-specific T cell subset in peripheral blood of melanoma patients [27]. Previous studies reported that PD-1^+^CD8^+^ T cells are enriched in PTC tumor-involved lymph nodes, and these cells were associated with dysfunction [10]. Based on the PD-1 expression levels, intermediate levels of PD-1^+^ stem-like CD8^+^ T cells are critical in maintaining T cell responses in conditions of antigen persistence [28]. Our flow cytometry analysis focused on total PD-1+ and terminally exhausted PD-1^hi^TIM-3^+^ CD8^+^ T cells. We did not quantify PD-1^int^ stem-like CD8^+^ T cells in this study; therefore, no conclusion can be drawn regarding their relative abundance in N0 versus N1 PTC. Increased numbers of PD-1^hi^TIM-3^+^ cells were positively associated with the presence of tumor cell extrathyroidal extension. Our data indicated that the tumor tissues of PTC with LNM carried a terminally exhausted immune feature.

These observations align with prior reports linking immune infiltration to tumor invasion and *BRAF*-driven immune remodeling. Kotwal et al. characterized spatial immune heterogeneity at the invasive leading edge of differentiated thyroid cancer, demonstrating that immune cell distribution varies across tumor microregions [17]. Our finding that terminally exhausted PD-1^hi^TIM-3^+^ CD8^+^ T cells are enriched in LNM-positive PTC and positively associated with extrathyroidal extension extends this concept by identifying a specific dysfunctional T cell subset that may underlie the invasive phenotype at the tumor margin. Means et al. reported that immune microenvironment characteristics in PTC correlate with histopathologic aggressiveness and *BRAF* mutation status [18]. Consistent with this, our TCGA analysis revealed that the terminal exhaustion signature was significantly enriched in *BRAF*-mutated tumors, and our clinical samples showed elevated IL-6 and CCL5 levels in LNM-positive PTC—cytokines known to be modulated by *BRAF*-driven signaling. Zhang et al. demonstrated that combined *BRAF* (*V600E*) and PD-L1 expression predicts clinicopathological aggressiveness and recurrence risk [19]. Our data complement this by showing that *BRAF* mutation is associated with terminal exhaustion signature enrichment rather than merely PD-L1 expression alone, suggesting that comprehensive immune exhaustion profiling may offer additional prognostic value beyond single checkpoint markers. Finally, Mediratta et al. confirmed in a meta-analysis that PD-L1 expression is associated with local invasion and distant metastasis in differentiated thyroid cancer [20]. Our findings expand this checkpoint-centric view by revealing concurrent upregulation of multiple inhibitory pathways (PD-1/TIM-3 on CD8^+^ T cells and CTLA-4 on CD4^+^ T cells) alongside Treg enrichment in LNM-prone PTC, indicating a broader immunosuppressive network that may cooperate to facilitate lymph node metastasis.

Regulatory T cells (Treg) are a subset of CD4^+^ T cells originally defined by the surface expression of high CD25 levels, low CD127 levels, and intracellular FOXP3. These cells are crucial for the maintenance of immunological self-tolerance by suppressing self-reactive T cells [29]. CTLA-4 is constitutively expressed by Treg cells and upregulated by CD8^+^ effector T cells after activation [24]. These cells are frequently increased in tumors. A previous report showed that the percentage of Tregs was increased in tumor and metastatic lymph nodes of patients with PTC [30]. In our current analysis, we detected an elevated frequency of phenotypically defined Treg cells among the total CD3^+^CD4^+^ T cells, and the elevation was significantly associated with LNM. While statistically significant, the modest difference in CTLA-4 MFI and the overlapping distributions should be interpreted cautiously given the limited sample size and exploratory nature of this study.

*BRAF* mutation (predominantly V600E) is a key driver of PTC aggressiveness, and our data link it to terminal immune exhaustion. Using data from TCGA-PTC, we found that *BRAF* gene mutation was associated with terminal exhaustion signature enrichment in tumors. *BRAF V600E* activates the MAPK pathway, which upregulates PD-L1 expression on tumor cells and induces T cell exhaustion [31,32]. Additionally, *BRAF V600E* may promote the secretion of IL-6 and CCL5, further amplifying the exhausted TME [33]. This finding suggests that *BRAF* mutation and terminal immune exhaustion may form a potential feed-forward loop associated with LNM, providing a rationale for combining *BRAF* inhibitors with anti-PD-1/TIM-3 therapy in high-risk PTC.

The results from our current study point to the association of T cell immune exhaustion with lymph node metastasis of PTC. However, further studies are required to validate the immunological character of PTC for predicting distant metastases and disease recurrence risk. Clinically, multiple types of cancer patients benefit from anti-PD-1 therapy. Our results also suggest a potential role for anti-PD-1 therapy in PTC patients carrying the specified immunological character, though this remains to be tested in prospective trials.

### 4.1. Clinical Implications

Our study addresses an unmet clinical need: preoperative identification of LNM-prone PTC. Although our measurements were performed on freshly resected tumor tissue and preoperative assessment remains to be validated, the terminal exhaustion signature (PD-1^hi^TIM-3^+^ CD8^+^ T cells, Treg frequency) could serve as a postoperative or investigational biomarker for risk stratification, potentially complementing imaging modalities. If validated in future preoperative biopsy studies, more extensive lymph node dissection may be warranted for patients with high exhaustion scores. Additionally, the enrichment of PD-1^hi^TIM-3^+^ cells in LNM-positive PTC suggests that anti-PD-1/TIM-3 combination therapy may be effective in these patients, particularly those with *BRAF* mutation.

### 4.2. Limitations

This study has several important limitations. First, the relatively small sample size (*n* = 40, with cytokine profiling in only 13 samples) limits statistical power and increases the risk of type I error and overinterpretation. No formal power analysis was conducted a priori; the study should be considered exploratory and hypothesis-generating rather than confirmatory. Second, due to the limited sample size, we were unable to perform multivariable logistic regression to adjust for potential confounders such as age, tumor size, *BRAF* status, thyroiditis, and extrathyroidal extension; therefore, whether terminal exhaustion independently predicts LNM remains unclear. Third, the TCGA analysis provides transcriptomic support consistent with the observed phenotype rather than direct validation, as it relies on gene expression signatures rather than direct measurements of immune-exhausted T cells. Moreover, the GSVA signature includes CD8A, GZMA, and PRF1, which may partly reflect overall CD8^+^ T cell infiltration or cytotoxic activity rather than terminal exhaustion specifically, and the modest absolute differences in GSVA scores should be interpreted with caution despite statistical significance in the large TCGA cohort. Fourth, the observational design cannot establish causality. Future prospective studies should validate the terminal exhaustion signature as a predictive biomarker and explore the efficacy of targeted therapies in high-exhaustion PTC. Fifth, Treg cells were phenotypically defined as CD3^+^CD4^+^CD25^hi^CD127^lo^ cells without intracellular FOXP3 confirmation, which may introduce phenotypic imprecision. Additionally, functional assays (e.g., cytokine production and cytotoxicity) would confirm the impairment of PD-1^hi^TIM-3^+^ CD8^+^ T cells. Finally, longer follow-up is needed to determine whether the terminal exhaustion phenotype correlates with recurrence and survival.

## 5. Conclusions

LNM-prone PTC is characterized by a terminal immune exhaustion phenotype, marked by PD-1^hi^TIM-3^+^ CD8^+^ T cell enrichment, Treg infiltration, and a pro-inflammatory immunosuppressive TME. This phenotype is conserved in the TCGA-PTC cohort and strongly correlates with *BRAF* mutation. These findings provide novel insights into the immunopathogenesis of PTC metastasis and identify potential biomarkers and therapeutic targets for high-risk patients.

## Figures and Tables

**Table 1:** Clinicopathological characteristics of the patients.

	N0 (*n* = 18)	N1 (*n* = 22)	*p*-Value
Gender			0.427
Male	2	5	
Female	16	17	
Age (Year) *	46 ± 13	36 ± 7	0.004
Histological subtype			0.11
Conventional	12	20	
Follicular variant	6	2	
Tumor size (mm) *	11.44 ± 4.48	13.41 ± 5.90	0.252
Thyroiditis			0.186
Yes	14	12	
No	4	10	
Benign nodules			0.341
Yes	11	9	
No	7	13	
Extrathyroidal extension			
Yes	13	18	0.705
No	5	4	

* Data are expressed as mean ± SD.

## Data Availability

All data supporting the conclusions of this study are included within the article and its supplementary Files. TCGA data are available from the GDC Portal (http://portal.gdc.cancer.gov/) on 20 June 2023.

## Supplementary Materials

The following supporting information can be downloaded at https://www.mdpi.com/article/10.3390/cancers18172873/s1, Table S1: List of antibodies.
