# Supplementary material for: Papillary Thyroid Carcinoma with Terminal Immune Exhaustion Phenotype Correlates with Increased Risk of Lymph Node Metastasis: An Exploratory Study Combining Flow Cytometry and TCGA
Source: Cancers (Basel). 2026 Sep 5;18(17):2873. doi: 10.3390/cancers18172873 (PMC13564956; doi:10.3390/cancers18172873)
Supplement: Supplementary file 1 [file cancers-18-02873-s001.zip › cancers-4493265-supplementary.pdf]

Table S1. List of antibodies

Antibody	Application	Manufacturer	Location	Cat.No.#	Dilution
CD45	IHC	Proteintech	Shanghai, China	60287-1-lg	1:5000
CD45	FCM	Biolegend	San Diego, CA, USA	304016	
CD3	FCM	Biolegend	San Diego, CA, USA	317342	
CD8	FCM	Biolegend	San Diego, CA, USA	300906	
PD-1	FCM	Biolegend	San Diego, CA, USA	329907	
TIM-3	FCM	Biolegend	San Diego, CA, USA	345007	
CD45	FCM	Biolegend	San Diego, CA, USA	304012	
CTLA-4	FCM	Biolegend	San Diego, CA, USA	369603	
CD127	FCM	Biolegend	San Diego, CA, USA	351309	
CD4	FCM	eBioscience	San Diego, CA, USA	11-0048-41	
CD8	FCM	eBioscience	San Diego, CA, USA	45-0088-41	
CD25	FCM	eBioscience	San Diego, CA, USA	25-0259-41	

IHC: Immunohistochemistry; FCM: Flow cytometry. #Cat. No. M500KCAF0Y, Bio-Rad Laboratories, Hercules, CA, USA
